# A study on the inhibitory mechanism for cholesterol absorption by α-cyclodextrin administration

**DOI:** 10.3762/bjoc.10.300

**Published:** 2014-12-02

**Authors:** Takahiro Furune, Naoko Ikuta, Yoshiyuki Ishida, Hinako Okamoto, Daisuke Nakata, Keiji Terao, Norihiro Sakamoto

**Affiliations:** 1 Division of Food and Drug Evaluation Science, Graduate School of Medicine, Kobe University, 650-0017, Japan; 2 CycloChem Bio Co., Ltd., 650-0047, Japan

**Keywords:** α-cyclodextrin, bile salt micelles, cholesterol, lecithin, micellar solubility

## Abstract

**Background:** Micelle formation of cholesterol with lecithin and bile salts is a key process for intestinal absorption of lipids. Some dietary fibers commonly used to reduce the lipid content in the body are thought to inhibit lipid absorption by binding to bile salts and decreasing the lipid solubility. Amongst these, α-cyclodextrin (α-CD) is reportedly one of the most powerful dietary fibers for decreasing blood cholesterol. However, it is difficult to believe that α-CD directly removes cholesterol because it has a very low affinity for cholesterol and its mechanism of action is less well understood than those of other dietary fibers. To identify this mechanism, we investigated the interaction of α-CD with lecithin and bile salts, which are essential components for the dissolution of cholesterol in the small intestine, and the effect of α-CD on micellar solubility of cholesterol.

**Results:** α-CD was added to Fed-State Simulated Intestinal Fluid (FeSSIF), and precipitation of a white solid was observed. Analytical data showed that the precipitate was a lecithin and α-CD complex with a molar ratio of 1:4 or 1:5. The micellar solubility of cholesterol in the mixture of FeSSIF and α-CD was investigated, and found to decrease through lecithin precipitation caused by the addition of α-CD, in a dose-dependent manner. Furthermore, each of several other water-soluble dietary fibers was added to the FeSSIF, and no precipitate was generated.

**Conclusion:** This study suggests that α-CD decreases the micellar solubility of cholesterol in the lumen of the small intestine via the precipitation of lecithin from bile salt micelles by complex formation with α-CD. It further indicates that the lecithin precipitation effect on the bile salt micelles by α-CD addition clearly differs from addition of other water-soluble dietary fibers. The decrease in micellar cholesterol solubility in the FeSSIF was the strongest with α-CD addition.

## Introduction

α-Cyclodextrin (α-CD) is a ring molecule composed of six glucose units that has an inclusion property with lipophilic molecules [1]. For example, α-CD has a high affinity for fatty acids, flavor molecules and other hydrophobic molecules [2–4]. However, α-CD has a low affinity for the steroid structure because the cavity size of α-CD is smaller than the structure [3,5]. α-CD has various effects on stabilization of fatty acids, flavor retention and emulsion formation of triglycerides via the formation of an inclusion complex [4,6–8].

α-CD is not used only as an encapsulation agent but also as a water-soluble dietary fiber. Furthermore, it has been reported that α-CD intake has beneficial effects on body weight control, lipid metabolism, glucose metabolism, prebiotics, allergy suppression and other functions [9–17]. It is thought that the mechanism behind the lowering of blood triglycerides by α-CD was the latter’s complexation with the fatty acid chains of the triglycerides, followed by the complex forming a stable emulsion [9]. However, the mechanism behind the decrease in blood cholesterol after α-CD administration remains unclear. Because α-CD has a very low affinity for cholesterol [3], it is difficult to believe that α-CD directly removes cholesterol through formation of an inclusion complex. Because α-CD is sparingly absorbed by the body [18], it is thought that α-CD acts within the lumen of the gut.

Lecithin and bile salts are major cholesterol-solubilizing agents found in gallbladder bile and form mixed micelles [19–21]. Formation of micelles comprising cholesterol, lecithin and bile salts is a very important process in enhancing cholesterol absorption from the lumen of the small intestine [22–23]. The ring size in α-CD is smaller than bile acids, so it has been reported that α-CD has a low affinity for bile acids [5,24–25]. Conversely, it has been reported that α-CD releases lecithin from the cell membrane [26–30].

In this study, we investigated the effect of α-CD on the mixed micelles (bile salt micelles) using Fed-State Simulated Intestinal Fluid (FeSSIF) [31] and the effect of α-CD on the micellar solubility of cholesterol in the FeSSIF. Furthermore, we compared several other water-soluble dietary fibers with α-CD to evaluate the effect of cholesterol micellar solubility in the FeSSIF.

## Results and Discussion

### α-CD precipitated with lecithin in the FeSSIF

#### α-CD generated a white precipitate in the FeSSIF

**Figure 1:**
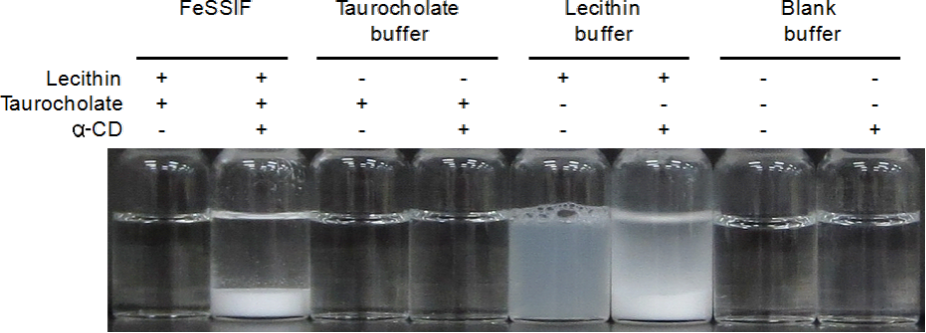
Image of the FeSSIF and other buffers with and without α-CD. α-CD was added into the FeSSIF or other buffers (37 °C) at a concentration of 5 w/v%. The taurocholate buffer was prepared using the same method as for FeSSIF preparation but without lecithin. The lecithin buffer was prepared using the same method as for the FeSSIF preparation but without sodium taurocholate. The blank buffer was prepared using the same method as FeSSIF preparation but both lecithin and sodium taurocholate were omitted.

α-CD was added into the FeSSIF to investigate their interactions. The composition of FeSSIF is shown in Table 1. Sodium taurocholate is a naturally occurring bile salt found in the human small intestinal fluid that is used preferentially for biorelevant dissolution testing [31]. FeSSIF alone was a clear solution with a light yellow color. An α-CD concentration of 5% generated a white precipitate in the FeSSIF (Figure 1) but no precipitate was generated in lecithin-free FeSSIF when the same amount of α-CD was added. These results probably indicate that lecithin was precipitated from FeSSIF by addition of α-CD.

**Table 1:** Composition of the Fed-State Simulated Intestinal Fluid (FeSSIF).

	mM

Sodium taurocholate	15
Lecithin	3.75
Acetic acid	144
Sodium chloride	173
Sodium hydroxide	~101
pH	5

#### α-CD precipitated with lecithin in the FeSSIF

**Figure 2:**
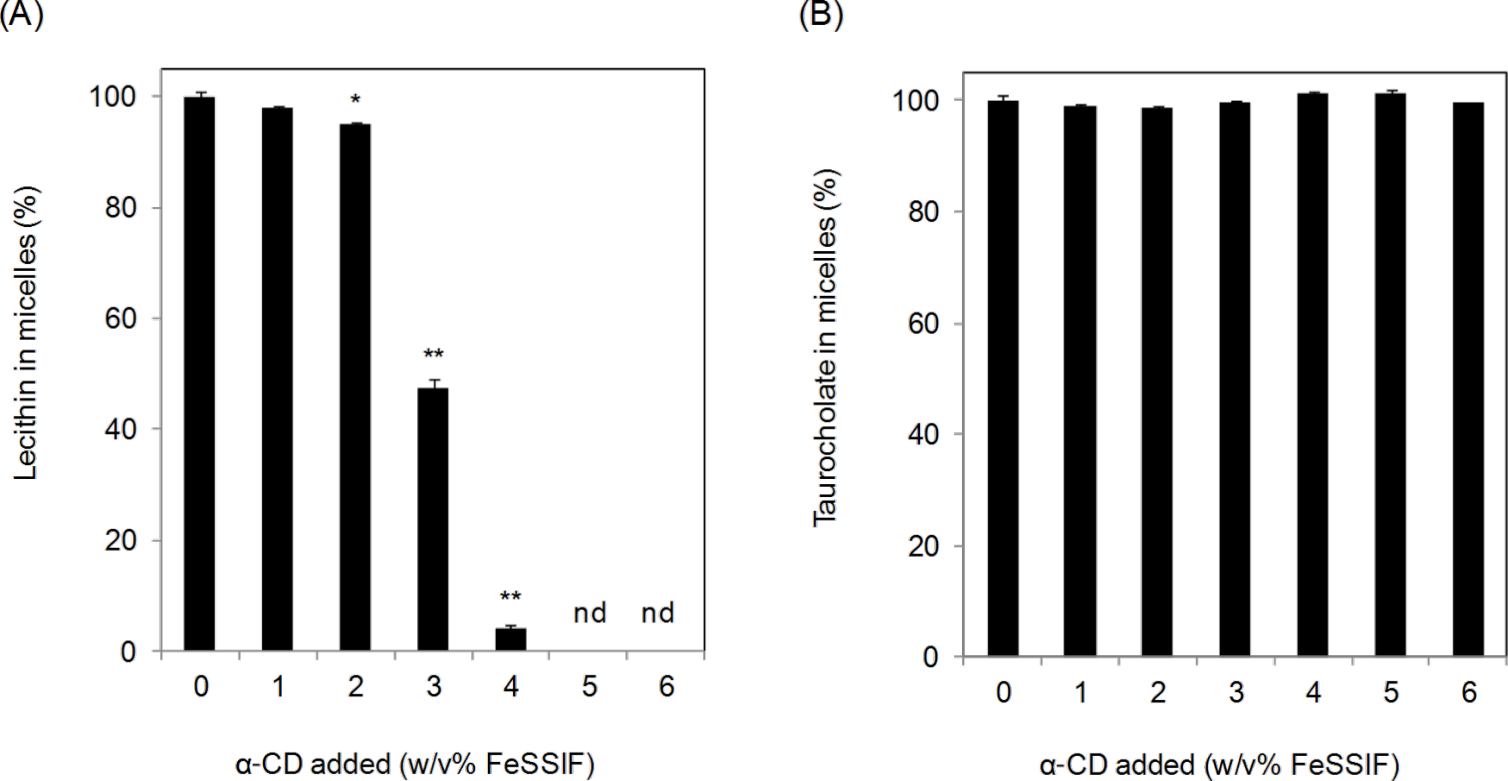
Effect of α-CD on the concentration of lecithin and taurocholate in the FeSSIF. After adding each amount of α-CD into 15 mL of FeSSIF (37 °C) and mixing for a few seconds, the mixture was shaken at 37 °C for 150 minutes at 100 rpm. The mixture was then filtered through a 0.2 μm PTFE filter. Lecithin (A) and taurocholate (B) contents in the filtrate were measured. Means and standard errors are indicated (*n* = 3). *P < 0.05, **P < 0.01, significantly different from α-CD 0%. nd: not detected.

To analyze the white precipitate, a mixture of FeSSIF and α-CD was shaken at an agitation rate of 100 rpm at 37 °C for 150 minutes and the lecithin, α-CD and taurocholate contents in a filtrate prepared from the mixture were quantified. The lecithin content was decreased by the addition of α-CD, following a reverse sigmoidal dose-response (Figure 2A). The lecithin content in the filtrate was clearly decreased by half through the addition of 3% α-CD, and eliminated completely when α-CD addition exceeded 5%. Although it has been reported that α-CD released lecithin from the cell membrane [26–30], this is the first time that the effect of α-CD on lecithin precipitation from bile salt micelles has been described. The taurocholate content in the filtrate was unaffected by the α-CD addition (Figure 2B). Because α-CD has a low affinity with taurocholate [5,24–25], this result was considered to be reasonable.

**Figure 3:**
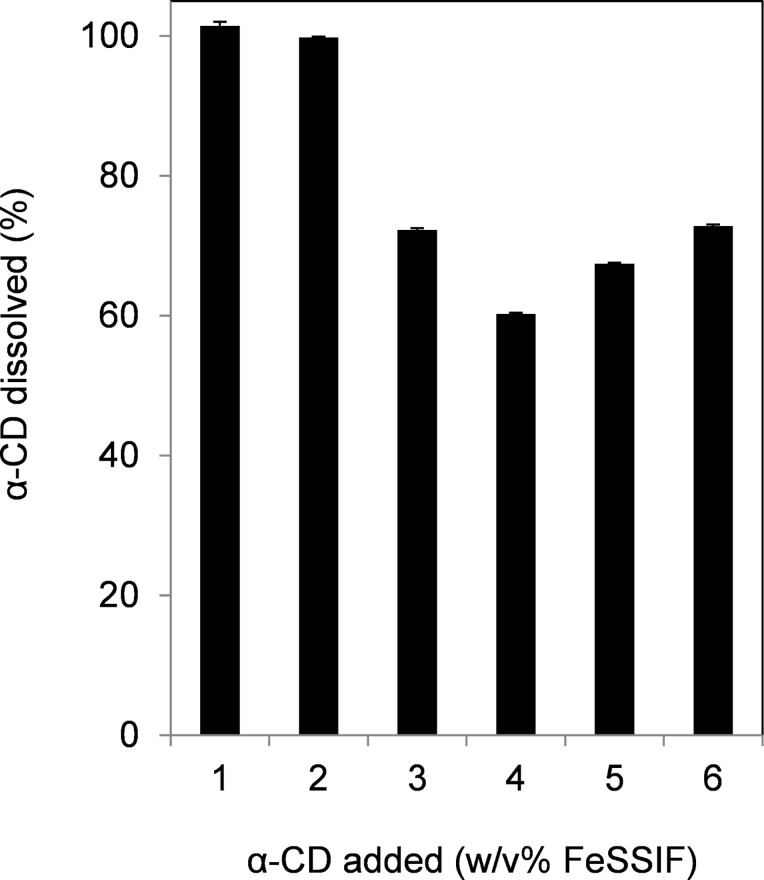
Concentration of α-CD in the FeSSIF. The experimental conditions were the same as those described in the legend to Figure 2. The α-CD content in micelles (%) are given as dissolved α-CD content compared with additional α-CD content. Means and standard errors are indicated (*n* = 3).

All α-CD dissolved in the FeSSIF at 1–2% addition (Figure 3). However, above 3% addition, the amount of α-CD dissolved was relatively low compared with the amount of α-CD added. The saturated solubility of α-CD in water was 20.4% at 35 °C [1], while 6% α-CD could be dissolved in the blank buffer. Further, precipitation also occurred when an aqueous solution of α-CD was added into the FeSSIF (data not shown). These results therefore indicate that α-CD precipitated in the FeSSIF.

**Figure 4:**
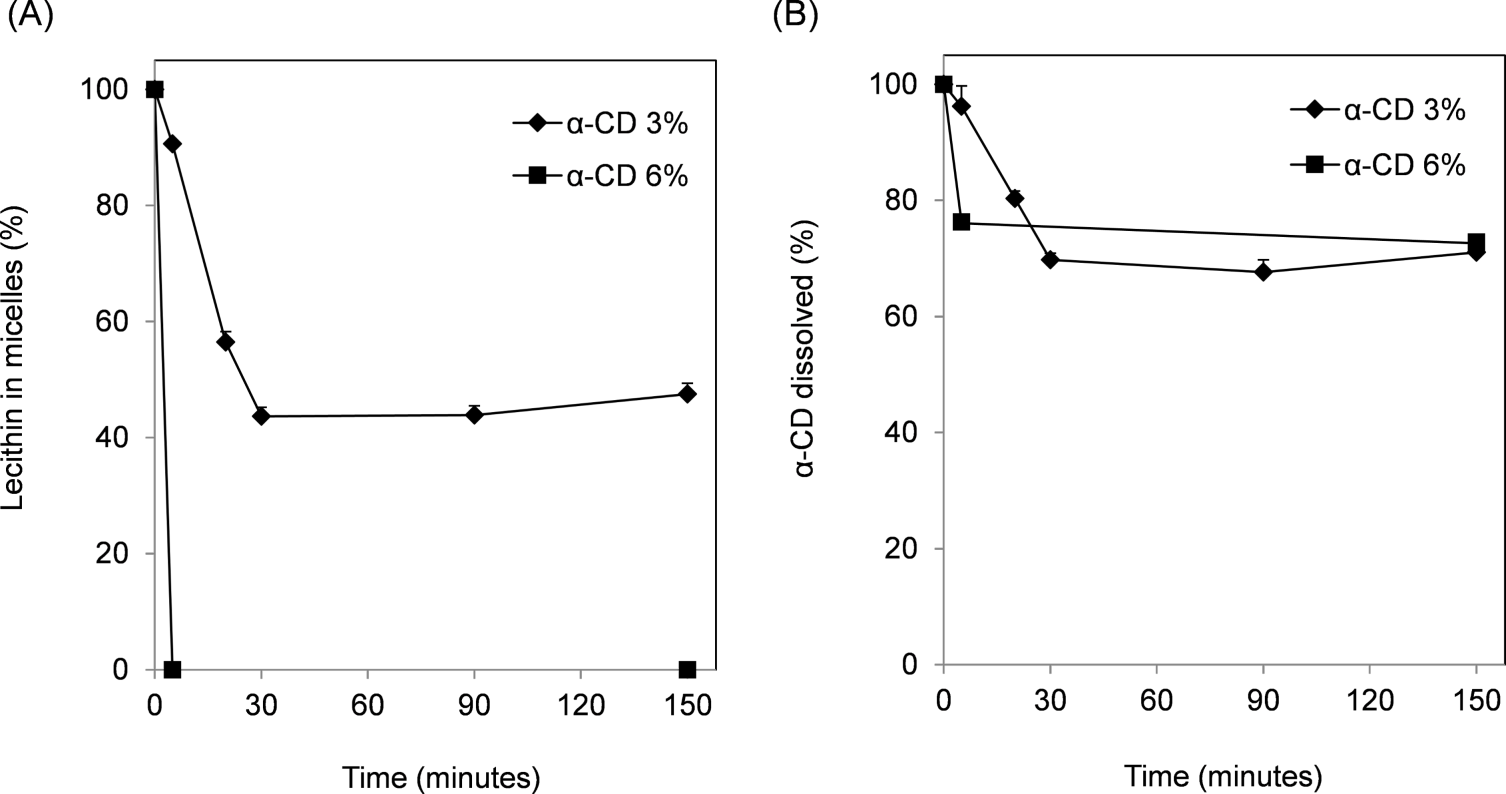
Time-dependent relationship between decreases in lecithin and α-CD. The experimental conditions were the same as those described in the legend to Figure 2, except for shaking time. Lecithin (A) and α-CD (B) contents in the filtrate were measured. α-CD in micelles (%) given as dissolved α-CD content compared with additional α-CD content. Means and standard errors are indicated (*n* = 3).

The relationship between the lecithin content and the length of shaking time after α-CD addition was investigated. The lecithin content decreased linearly until 30 minutes after addition of α-CD (3%) into the FeSSIF and then remained constant up to 150 minutes (Figure 4A). At 6% α-CD, the lecithin was eliminated within 5 minutes. The time course for α-CD precipitation correlated with the time course for lecithin decrease (Figure 4B). These results indicate the co-precipitation of lecithin and α-CD, suggesting that lecithin and α-CD formed a complex.

#### Molar ratio of lecithin and α-CD of the white precipitate was 1:4.4

**Figure 5:**
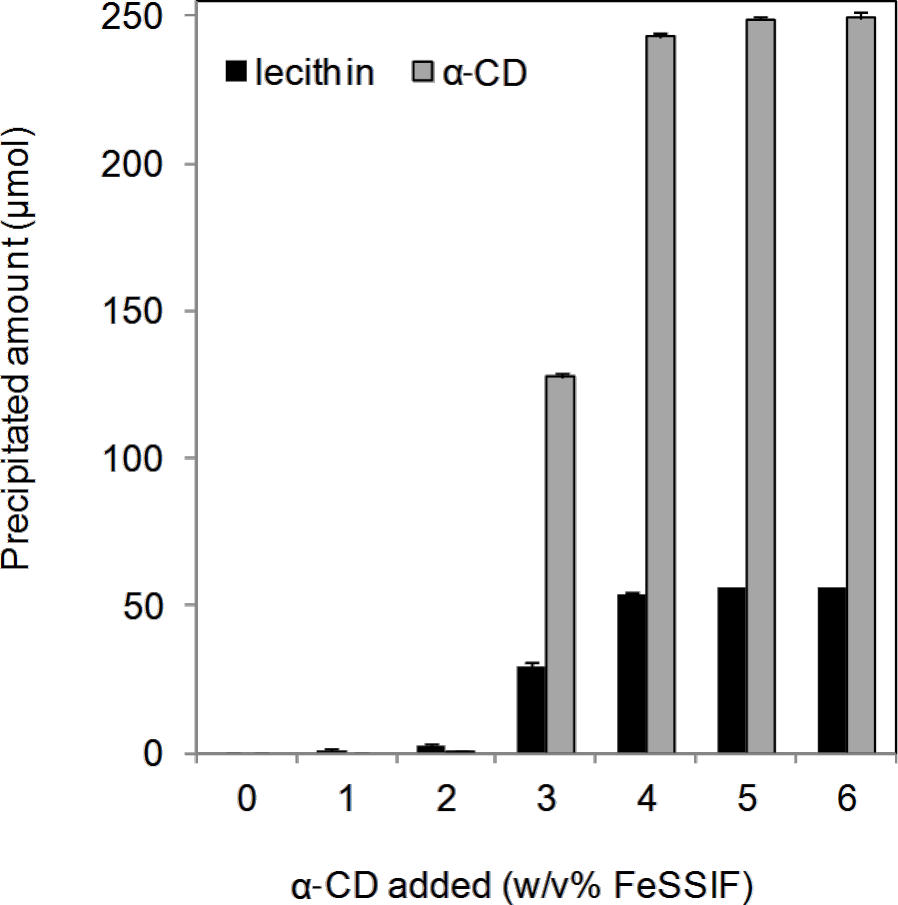
Amounts of lecithin and α-CD precipitates. The amounts of lecithin and α-CD precipitated were calculated from the data shown in Figure 2A and Figure 3, respectively. Means and standard errors are indicated (*n* = 3).

To investigate the ratio of lecithin and α-CD within the precipitate, the amounts of precipitated lecithin and α-CD were calculated from the data in Figure 2A and Figure 3, respectively (Figure 5). The precipitated lecithin and α-CD increased with the addition of α-CD in a sigmoidal dose-response curve. The amounts of lecithin and α-CD precipitated did not increase in the FeSSIF containing over 5% α-CD.

The molar ratio of lecithin and α-CD in the precipitate corresponded to around 1:4.4 (1:4.3 (α-CD 3%), 1:4.5 (α-CD 4%), 1:4.4 (α-CD 5%), 1:4.4 (α-CD 6%), stated as the ratios of lecithin to α-CD). Because Schlenk et al. reported that one mol of C_16_–C_18_ fatty acid was complexed with three mol of α-CD [2], it is possible that (1-palmitoyl-2-oleoyl)lecithin, a major component of lecithin [32], was complexed with up to six α-CD molecules. These results suggest that the two fatty acids of lecithin were complexed with four or five α-CD molecules.

### α-CD decreased the micellar solubility of cholesterol via lecithin precipitation

**Figure 6:**
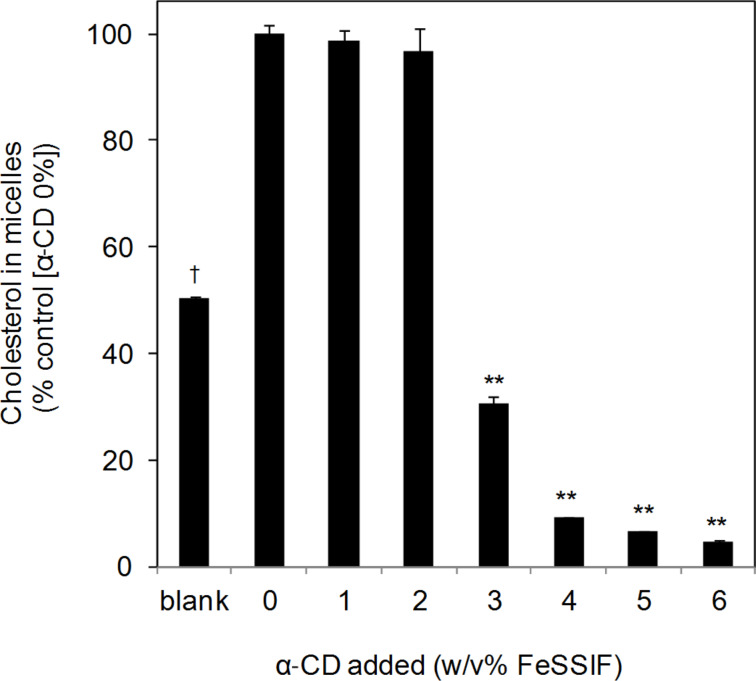
Dose-dependent decrease of the micellar cholesterol solubility in the FeSSIF by α-CD. After addition of each amount of α-CD into 15 mL of the FeSSIF or an alternative buffer (37 °C) and mixing for a few seconds, the mixture was shaken at 37 °C for 30 minutes at 100 rpm. Thirty milligrams of cholesterol was then added into the mixture, and the mixture was shaken for 120 minutes at 37 °C at 100 rpm. After centrifuging the mixture for 10 minutes at 10,000 rpm, the supernatant was filtered using a 0.2 μm PTFE filter. The blank value gives the amount of cholesterol originally dissolved in the FeSSIF. Means and standard errors are indicated (*n* = 3). **^,†^*P* < 0.01, significantly different from control (α-CD 0%).

α-CD decreased the micellar solubility of cholesterol in a dose-dependent manner (Figure 6). The blank value gives the original concentration of dissolved cholesterol in the FeSSIF, in the absence of cholesterol addition, and was derived from impurity of the lecithin reagent used. The cholesterol concentration dissolved within the FeSSIF increased with cholesterol addition. The addition of 1 or 2% α-CD into the FeSSIF did not affect the cholesterol dissolution in the FeSSIF but the addition of 3% α-CD decreased the dissolved cholesterol concentration to 30% of that of the control. The addition of 4% α-CD into the FeSSIF reduced the dissolved cholesterol concentration below 10% of that of the control.

The micellar cholesterol solubility corresponded to the lecithin precipitation. The observed relationship between the micellar cholesterol solubility and the lecithin content in the bile salt micelles is consistent with the report of Kobayashi et al. [33]. Because α-CD has a very low affinity for cholesterol [3], it is suggested that the decrease in micellar cholesterol solubility in the FeSSIF was mainly caused by lecithin precipitation through interaction with α-CD. Furthermore, because the human bile contains bile salts, lecithin and cholesterol [22,34], these results suggest that α-CD may decrease not only the cholesterol in foods but also the cholesterol originally dissolved in human intestinal fluid.

### Comparison of micellar cholesterol solubility after addition of several water-soluble dietary fibers

We compared several water-soluble dietary fibers with α-CD to evaluate their effects on micellar cholesterol solubility in the FeSSIF. Five dietary fibers with a lipid lowering effect were chosen and all tested fibers were commercially available in Japan (Resistant Maltodextrin, RM; Partially Hydrolyzed Guar Gum, PHGG; Inulin, Inu; Polydextrose, PDX [35–40]). Cholestyramine (CSA) was used as a positive control. The CSA has the ability to bind bile salts [41] and is used to lower blood cholesterol levels.

**Figure 7:**
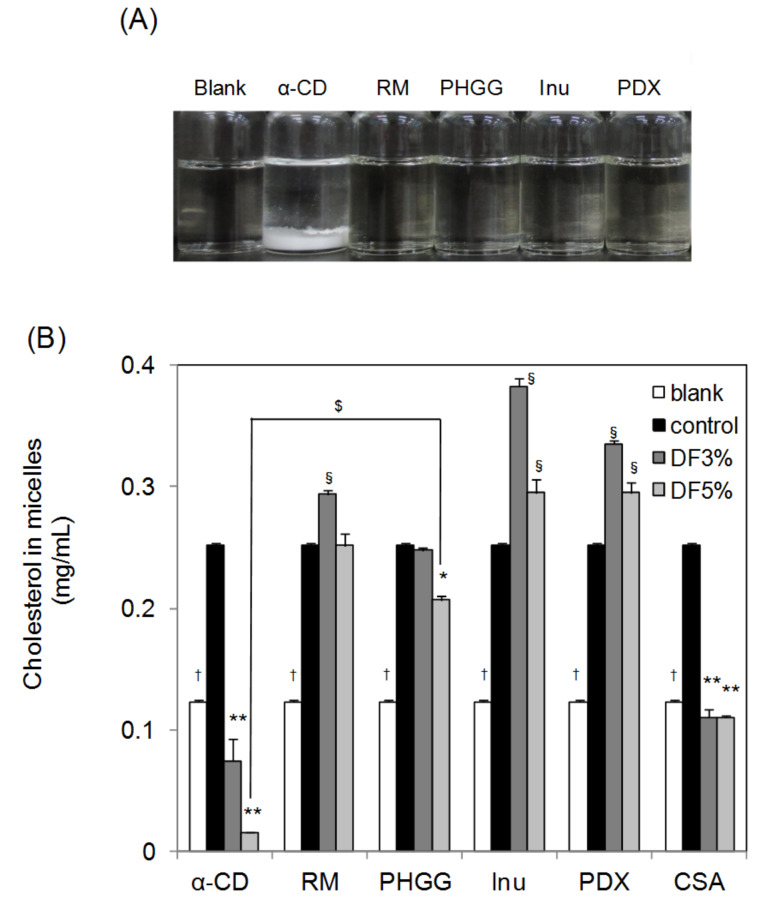
Effect of several dietary fibers on the micellar cholesterol solubility in FeSSIF. Various amounts of α-CD, Resistant Maltodextrin (RM), Partially Hydrolyzed Guar Gum (PHGG), Inulin (Inu), Polydextrose (PDX) or Cholestyramine (CSA) were added to 15 mL of FeSSIF at 37 °C. The experimental conditions were the same as those described in the legend to Figure 6. (A) Image of FeSSIF with each dietary fiber at a concentration of 5%. (B) Cholesterol concentration in micelles after shaking for 120 minutes with additional cholesterol (control) or with no added α-CD or cholesterol (blank). Means and standard errors are indicated (*n* = 3). **^,†,§^*P* < 0.01, significantly different from control. ^$^*P* < 0.01, significantly different between α-CD 5% and PHGG 5%.

No precipitate was generated in the FeSSIF by addition of water-soluble dietary fibers, apart from α-CD at 37 °C (Figure 7A). CSA was insoluble in water and FeSSIF. α-CD, PHGG and CSA decreased the micellar solubility of cholesterol, in contrast to the other dietary fibers (Figure 7B). These results show that α-CD was the most effective water-soluble dietary fiber with respect to decreasing micellar cholesterol solubility in the FeSSIF, because only α-CD readily formed a complex with lecithin.

At a 5% addition of PHGG, micellar cholesterol solubility in FeSSIF was lower than the control. It has been reported that PHGG suppresses postprandial triglyceride elevation and decreases the bioaccessibility of cholesterol through the depletion flocculation mechanism [35–36]. The effect of PHGG on the micellar cholesterol solubility in this study is consistent with the results of Minekus et al. [36]. RM did not decrease micellar cholesterol solubility in FeSSIF. It has been reported that RM decreases postprandial triglyceride elevation, and it was suggested that the mechanism was the stabilization of mixed micelles of fatty acids, fatty acid esters and bile salts [37–38]. Thus, the mechanism behind RM showing a higher micellar cholesterol solubility than the control is considered to be related to its micelle stabilization function. Conversely, although Inu and PDX were reported to lower lipid contents [39–40], our current study shows that these dietary fibers did not decrease micellar solubility of cholesterol.

These results indicate that α-CD is a unique water-soluble dietary fiber because it operates by a different mechanism towards micellar cholesterol solubility than other water-soluble dietary fibers, and α-CD was the most effective amongst the water-soluble dietary fibers tested.

### Putative mechanism for inhibition of cholesterol absorption by α-CD

**Figure 8:**
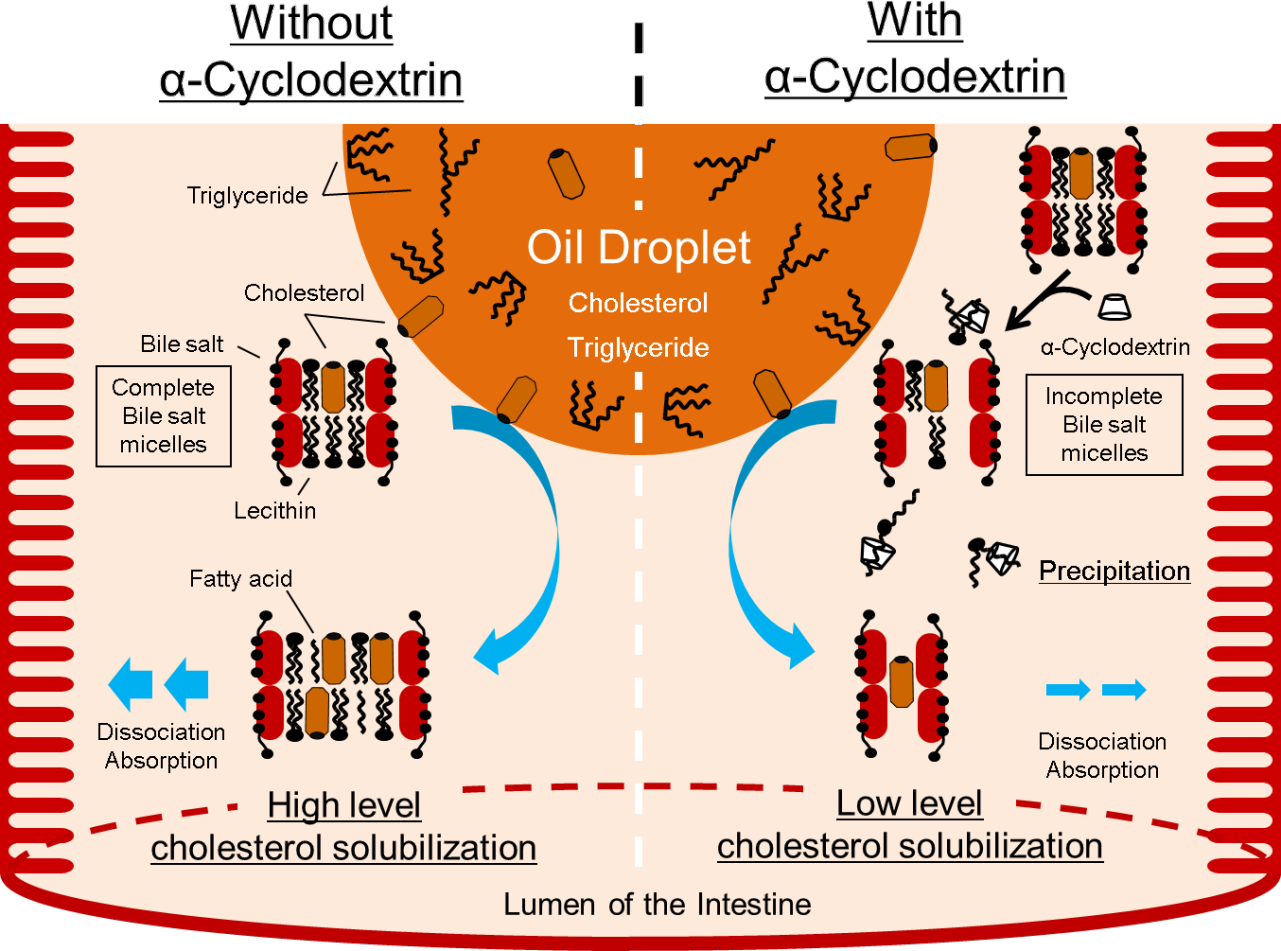
Hypothetical scheme for the inhibitory action of α-CD on the micellar cholesterol solubility in intestinal fluid. α-CD precipitates lecithin from bile salt micelles within the lumen of the small intestine and thereby indirectly decreases the micellar solubility of cholesterol.

The addition of α-CD to the FeSSIF decreased the lecithin content (Figure 2A). This result suggests that lecithin and α-CD formed a complex and caused formation of insolubles. α-CD decreased the micellar solubility of cholesterol in a dose-dependent manner (Figure 6). The decrease in micellar cholesterol solubility in the FeSSIF was caused mainly by lecithin precipitation through its interaction with α-CD. This study indicates that orally-ingested α-CD can precipitate lecithin from the bile salt micelles within the lumen of the small intestine and thus indirectly decreases the micellar solubility of cholesterol (Figure 8, right side).

We investigated other water-soluble dietary fibers and found that α-CD is unique in that it affects micellar cholesterol solubility by a different mechanism. α-CD was the most effective amongst the water-soluble dietary fibers we tested (Figure 7).

## Conclusion

Orally-ingested α-CD has a blood cholesterol lowering effect, even though α-CD is only sparingly absorbed in the body. It is thought unlikely that α-CD directly removes cholesterol because α-CD has a very low affinity for cholesterol. We found that lecithin was dose-dependently precipitated through the addition of α-CD to FeSSIF. The molar ratio of the precipitate indicates that lecithin and α-CD form a complex with a molar ratio of 1:4 or 1:5. α-CD decreases the micellar solubility of cholesterol via lecithin precipitation from the FeSSIF. Furthermore, we compared the addition of several water-soluble dietary fibers with that of α-CD to evaluate their effects on micellar cholesterol solubility in FeSSIF. Only α-CD generated a precipitate from FeSSIF. Both α-CD and PHGG decreased micellar cholesterol solubility but α-CD was the most effective of all water-soluble dietary fibers tested.

## Experimental

### Materials

α-CD was supplied by CycloChem (Kobe, Japan). Lecithin from hen’s egg (>95% pure) was purchased from Wako Pure Chemical Industries (Osaka, Japan). Sodium taurocholate, cholesterol, sodium chloride, sodium dihydrogen phosphate, disodium hydrogen phosphate, sodium hydroxide and acetic acid (each Wako special grade) were purchased from Wako Pure Chemical Industries (Osaka, Japan). Cholestyramine was purchased from Sigma-Aldrich Japan (Tokyo, Japan). Dietary fibers used in this study were resistant maltodextrin (Matsutani Chemical Industry, Hyogo, Japan), partially hydrolyzed guar gum (Taiyo Kagaku, Mie, Japan), inulin (Fuji Nihon Seito, Tokyo, Japan) and polydextrose (Koyo Mercantile, Tokyo, Japan).

### Equipment

A shaking water bath (model NTS-4000B; Tokyo Rikakikai, Tokyo, Japan) was used to shake test solutions. The HPLC instrument was a Shimadzu LC-2010C HPLC System (Shimadzu, Kyoto, Japan), a refractive index detector model RID-10A (Shimadzu, Kyoto, Japan) and a system controller SCL-10A VP (Shimadzu, Kyoto, Japan). Another HPLC instrument used was a Shimadzu LCMS-2020 system, with a reservoir section valve FCV-11AL and a valve unit FCV-20AH_2_ (Shimadzu, Kyoto, Japan)_._ The UV–vis spectrophotometer was a UV mini-1240 (Shimadzu, Kyoto, Japan). Minispin (eppendorf, Tokyo, Japan) was used for a centrifugation at 10,000 rpm. Model CN-1050 (MATSUURASEISAKUSYO, Tokyo, Japan) was used for a centrifugation at 3,000 rpm.

### Methods

#### Preparation of Fed-State Simulated Intestinal Fluid (FeSSIF)

The FeSSIF was prepared according to the method of Vertzoni et al. [31]. The taurocholate buffer was prepared using the same method as for FeSSIF preparation but lecithin was omitted. The lecithin buffer was prepared using the same method as for FeSSIF preparation but sodium taurocholate was omitted. The blank buffer was prepared using the same method as for FeSSIF preparation but both lecithin and sodium taurocholate were omitted.

#### Interaction study between α-CD and FeSSIF

After adding each amount of α-CD into 15 mL of the FeSSIF (37 °C) and mixing for a few seconds, the mixture was shaken at 37 °C at an agitation rate of 100 rpm. The mixture was then filtered through a 0.2 μm PTFE filter and the filtrate was diluted 1/10 using MilliQ water. The α-CD and taurocholate concentrations in the test solution were analyzed using HPLC. The lecithin concentration in the test solution was analyzed using LabAssay^TM^ Phospholipid (Wako Pure Chemical Industries, Osaka, Japan).

#### Solubility studies of cholesterol in FeSSIF

After adding each amount of α-CD into 15 mL of the FeSSIF (37 °C) and mixing for a few seconds, the mixture was shaken at 37 °C for 30 minutes at 100 rpm. 30 mg of cholesterol was then added into the mixture, and the mixture was shaken again at 100 rpm and 37 °C. After centrifuging the mixture for 10 minutes at 10,000 rpm, the supernatant was filtered using a 0.2 μm PTFE filter. An equal amount of ethyl acetate was added to the filtrate and mixed for 30 seconds using a vortex mixer. After centrifuging for 15 min at 3,000 rpm, the ethyl acetate layer was filtered through a 0.2 μm PTFE filter and the filtrate was analyzed by HPLC. A control solution was prepared using the same procedure but without the addition of α-CD.

#### Analytical methods of α-CD, taurocholate, lecithin and cholesterol

**HPLC conditions for α-CD:** The analytical column was an X Bridge^TM^ Amide (4.6 mm × 150 mm, Nihon Waters, Tokyo, Japan). The column temperature was 35 °C. The mobile phase was a mixture of acetonitrile and water (70:30, v/v). The flow rate was 0.8 mL/min. A refractive index detector was used. A limit of detection of 11 μg mL^−1^ was obtained at a signal-to-noise ratio of 3.

**HPLC conditions for taurocholate:** The concentration of taurocholate was measured by HPLC according to a method described by Shaw et al. [42]. An analytical column NUCLEOSIL 7C18 column (4.0 mm × 250 mm, Chemco Plus Scientific, Osaka, Japan) was used at 35 °C. The mobile phase was a mixture of 2-propanol and 8.8 mM potassium phosphate buffer pH 2.5 (160:340, v/v). The flow rate was 0.3 mL/min. UV detection was carried out at 210 nm. A limit of detection of 1.2 μg mL^−1^ was obtained at a signal-to-noise ratio of 3.

**Lecithin measurement:** The concentration of lecithin was determined as total lecithin using LabAssay^TM^ Phospholipid. Sample solution (0.5 mL) was added into 1.5 mL of a color reagent and thoroughly mixed. After incubation at 37 °C for 5 minutes, the absorbance at 600 nm was measured. A limit of detection of 3.75 μM was obtained at a signal-to-noise ratio of 3.

**HPLC conditions for cholesterol:** The concentration of cholesterol was determined by HPLC according to a method described by Zhang et al. [43]. The analytical column was a Sunfire C18 (4.6 mm × 150 mm, Nihon Waters, Tokyo, Japan). The column temperature was 40 °C. The mobile phase was a mixture of acetonitrile and isopropyl alcohol (8:2, v/v). The flow rate was 0.6 mL/min. UV detection was carried out at 208 nm. A limit of detection of 0.15 μg mL^−1^ was obtained at a signal-to-noise ratio of 3.

#### Statistical Analyses

Data are presented as means ± standard errors. Data were calculated using an ANOVA with Dunnett test to evaluate significant differences between pairs of means.
